# Combining Exergame Training with Omega-3 Fatty Acid Supplementation: Protocol for a Randomized Controlled Study Assessing the Effect on Neuronal Structure/Function in the Elderly Brain

**DOI:** 10.3389/fnagi.2016.00283

**Published:** 2016-11-29

**Authors:** Alexandra Schättin, Eling D. de Bruin

**Affiliations:** Department of Health Sciences and Technology, Institute of Human Movement Sciences and Sport, ETH ZurichZurich, Switzerland

**Keywords:** older adults, exergame training, video game, nutritional supplementation, omega-3 fatty acid, brain function, brain structure

## Abstract

A common problem in the older population is the risk of falling and related injury, immobility, and reduced survival. Age-related neuronal changes, e.g., decline in gray-and white-matter, affect neuronal, cognitive, and motor functioning. The improvement of these factors might decrease fall events in elderly. Studies showed that administration of video game-based physical exercise, a so-called exergame, or omega-3 fatty acid (FA) may improve motor and/or cognitive functioning through neuronal changes in the brain of older adults. The aim of this study is to assess the effects of a combination of exergame training with omega-3 FA supplementation on the elderly brain. We hypothesize that an intervention using a combination approach differently affects on the neuronal structure and function of the elderly's brain as compared to the sole administration of exergame training. The study is a parallel, double-blinded, randomized controlled trial lasting 26 weeks. Sixty autonomous living, non-smoking, and right-handed healthy older (>65 years) adults who live independently or in a senior residency are included, randomized, and allocated to one of two study groups. The experimental group receives a daily amount of 13.5 ml fish oil (including 2.9 g of omega-3 FA), whereas the control group receives a daily amount of 13.5 ml olive oil for 26 weeks. After 16 weeks, both groups start with an exergame training program three times per week. Measurements are performed on three time-points by treatment blinded investigators: pre-intervention measurements, blood sample after 16 week, and post-intervention measurements. The main outcomes are motor evoked potentials of the right M. tibialis anterior (transcranial magnetic stimulation) and response-related potentials (electroencephalography) during a cognitive test. For secondary outcomes, reaction time during cognitive tests and spatio-temporal parameters during gait performance are measured. Statistics will include effect sizes and a 2 × 2-ANOVA with normally distributed data or the non-parametric equivalent for data not fulfilling normal distribution. The randomized controlled study is the first to investigate the effectiveness of exergame training combined with omega-3 FA in counteracting age- and behavioral-dependent neuronal changes in the brain. This study has been registered in the Swiss National Clinical Trials (SNCTP000001623) and the ISRCTN (ISRCTN12084831) Portals.

## Introduction

In general, the human brain undergoes age-dependent changes by losing about 15% of the cerebral cortex and about 25% of the cerebral white-matter between the ages of 30 and 90 years (Colcombe et al., 2003). Age-associated alterations in gray-matter and white-matter integrity (Longstreth et al., 1996; Gunning-Dixon et al., 2009; Fjell and Walhovd, 2010) and a decrease in synthesis and binding of dopamine (produced in substantia nigra and ventral tegmental area), serotonin (produced in raphe nuclei), and acetylcholine (produced in pedunculopothine nucleus and laterodorsal tegmental nucleus, medial septal and diagonal band nuclei, and nucleus basalis; Wang et al., 1995, 1998; Volkow et al., 1998; Bäckman et al., 2006; Schliebs and Arendt, 2011) are connected to deteriorations of cognitive functioning, e.g., working memory and executive function (EF). EFs are interrelated cognitive abilities that control and guide goal-directed actions (Banich et al., 2009); e.g., walking in challenging environments. Different EF components, e.g., “working memory” (Holtzer et al., 2006), “divided attention” (Sheridan et al., 2003), and “inhibition” (Hausdorff et al., 2005), partly explain gait performance. Especially spatial and temporal dual-task cost characteristics of gait are associated with divided attention (de Bruin and Schmidt, 2010). Gait disturbances and fall events, caused by sensory and motor impairments, are believed to be moderated by executive functioning (Rapport et al., 1998; Scherder et al., 2011). Training of EFs in older adults might contribute to improved gait performance (Pichierri et al., 2012a) and might reduce fall events as EF performance predicted the risk for future falls (Mirelman et al., 2012). However, so far no direct cause and effect relationship was demonstrated between EF and gait (Yogev-Seligmann et al., 2008).

Anatomically, EFs have been linked with the frontal lobe of the brain, in particular the dorsolateral prefrontal cortex (PFC) and related brain networks (DeLong, 2000; Yogev-Seligmann et al., 2008). A large PFC volume and a greater PFC thickness were associated with better EFs (Yuan and Raz, 2014). During lifetime, the (pre) frontal network undergoes age-dependent neuronal changes; however, no consensus exists to the precise pattern of EF altering (Gunning-Dixon and Raz, 2003; Brickman et al., 2006; Yogev-Seligmann et al., 2008; Gunning-Dixon et al., 2009). One assumption is that the decline in frontal gray-matter might be associated with the deterioration of EFs (Zimmerman et al., 2006). Moreover, disturbances in cortico-cortical and cortico-subcortical connections, e.g., frontal connections with parietal lobes and basal ganglia, respectively, are classified as higher level gait disorders (Thompson and Nutt, 2007; Scherder et al., 2011). A phenomenon coined “retrogenesis” implies that brain circuits that mature late in ontogeny are most vulnerable to early neurodegeneration (Davis et al., 2009) and might contribute to the understanding and prediction of disturbances in higher level gait and gait-related motor activity. This suggestion is supported by recent work of Rosano et al. (2012) showing that a smaller volume of the prefrontal area is likely to contribute to slower gait through slower information processing (Rosano et al., 2012).

So far, training of cognitive abilities (e.g., EFs) may represent an important strategy to preserve brain function and also prevent mobility disability (de Bruin et al., 2011, 2013; Pichierri et al., 2012b; Rosano et al., 2012). Furthermore, recent reviews focusing on the interplay between physical function and cognition concluded that it seems important to combine motor and cognitive training into clinical practice to enable older adults to move safer in their physical environment (Segev-Jacubovski et al., 2011; Pichierri et al., 2012b; Bamidis et al., 2014). Especially, computerized interventions seem promising (Green and Bavelier, 2008; Pichierri et al., 2012b; Bamidis et al., 2014) when considering training principles that enhance (motor) learning (Green and Bavelier, 2008). Video games might have the potential to train cognitive functions (Zelinski and Reyes, 2009). A video game-based physical exercise, or a so-called exergame, allows the recommended combinatory training of motor and cognitive abilities. It is believed that physical exercise (PE) interventions with decision-making opportunities might facilitate the development of both motor performance and cognitive function (Yan and Zhou, 2009).

Recent research indicates that the effects of PE on the brain can be enhanced by concurrent consumption of natural products (van Praag, 2009). This means it can be hypothesized that a combination of physical training with a nutritional supplement (NS) has the potential to further enhance the effects of physical training on the level of brain structure and function in older persons. The potential synergy between nutrition and PE could involve common cellular pathways important for neurogenesis, cell survival, synaptic plasticity, and vascular function (van Praag, 2009).

A systematic review revealed that previous interventions using a combined approach of PE and NS to effect on the brain were not particularly successful because of the misfit between the combinations; the intervention components were not selected based on sharing of similar neuronal mechanisms (Schättin et al., 2016). The review indicates, however, that especially fish oil, containing omega-3 fatty acid (FA), might be an effective NS supporting the positive effects of PE. Omega-3 FA is important for energy metabolism and for the composition of the plasma membranes in the brain (Gómez-Pinilla, 2008). Another review showed that long chain polyunsaturated FA (LCPUFA) might improve cognition, decrease (neuro) inflammation, and reduce vascular risk factors in normal aging adults (Janssen and Kiliaan, 2014). Omega-3 LCPUFA may provide decreased brain deterioration through the positive effects on brain structure, function, and cerebral blood flow (Haast and Kiliaan, 2015). A recent randomized-controlled study showed that fish oil had positive effects on brain structure and function in healthy older adults (Witte et al., 2014). The participants showed improved EFs, white matter microstructure integrity, gray matter volume, and vascular parameters.

So far no study investigated the combined effect of exergame training and omega-3 FA on the elderly brain's structure and function. This study, therefore, aims to investigate the effects of a combination of exergame training and omega-3 FA. The following research question will guide through the research process: “Does the combination of exergame training and fish oil differently affect neuronal system levels in the elderly brain compared to exergame training alone?” The main objectives of the trial are (1) to determine the effects of the intervention on the neuronal structural level of the brain (neuronal excitability) and (2) to assess the effects on functional level in the brain (neuronal activity). We hypothesize that the combination will differently affect these parameters.

## Materials and methods

### Ethics and reporting

The study procedure has been approved by the local ethics committee (EC Zurich Switzerland, EC number: 2015-0190) and conforms to the Declaration of Helsinki and the guidelines of Good Clinical Practice E6 (R1). No data was recorded before written informed consent was given by the participants. The trial protocol follows the Consolidated Standards of Reporting Trials (CONSORT) statement on randomized trials of non-pharmacological treatment (Boutron et al., 2008) and Standard Protocol Items: Recommendations for Interventional Trials (SPIRIT) guidance for protocol reporting (Chan et al., 2013).

### Design and setting

The study is a randomized double-blinded, placebo-controlled study involving elderly adults above 65 years. The study is designed to examine the effect of omega-3 FA supplementation and exergame training on the endpoints of neuronal structure and function before and after a 26-weeks intervention period. The measurements and data collection, the exergame training, and data analysis are conducted at the same study site (ETH Hönggerberg, Zurich, Switzerland). At home, the participants are expected to take the NS regularly.

### Blinding, randomization, and allocation

The NS, packed in bottles equal in outer appearance, were blinded by an external center (Kantonsapotheke Zurich, Switzerland) to achieve double blinding. The external center created a computer-generated list including numbers from 001 to 060 that correspond to either fish oil or olive oil, respectively. The list number does not correspond to the participants' identification (ID) number. The list consists of six blocks of ten whereas fish oil and olive oil are randomly and equally distributed in all blocks. The investigators continuously assigned the volunteering women to the numbers starting with 001 and ending with 030 and the men starting with 031 and ending with 060. The randomization list is stored by a non-involved investigator and out of reach and sight of the involved investigators. For statistics, the groups will be referred to without specification of NS (e.g., group A and B).

### Participants

Participants were recruited from the Senior's University Zurich (Switzerland), senior residency dwellings in Zurich (Switzerland), and through public advertisement. The public advertisement included a brief study description and study site contact information. All those who were interested received a study information sheet including the design, procedure, benefits, and risks of the study. Before the study procedure started, the participants had to provide signed written informed consent forms. Participants fulfilling all of the following inclusion criteria were eligible for the study: (1) age above 65 years, (2) live independently or in a residency dwelling, (3), non-smoker, and (4) healthy (self-reported). Participants were excluded if they exhibited one of the following exclusion criteria: (1) mobility impairments, (2) orthopedic or neurological diseases that prevent training participation, (3) rapidly progressive or terminal illness as well as acute or chronic illness, (4) history of heart attack, stroke, or epilepsy, (5) medication that interacts with NS (e.g., hypoglycemic medication and anticoagulants), (6) medication that acts on neuronal level (e.g., psychotropic medications), (7) cognitive impairment (Mini Mental Status Examination <22 points), (8) signs of an upcoming depression (Geriatric Depression Scale), (9) electronic or metallic head implants, and (10) personal history of dizziness.

### Interventions

The study interventions are described in detail according to the Template for Intervention Description and Replication (TIDieR) guidelines (Hoffmann et al., 2014) in Table 1 to allow readers and other researchers to use or replicate the intervention.

**Table 1:** **Description of study intervention based on the Template for Intervention Description and Replication (TIDieR) checklist (Hoffmann et al., 2014)**.

**Item**	**Experimental group**	**Control group**
1. Brief name	Fish oil + exergame training	Olive oil + exergame training
2. Why?	Exergame training (Bamidis et al., 2014) as well as omega-3 FA (Witte et al., 2014) have positive effects on the elderly brain. The combination of exergame training and omega-3 FA might improve brain structure and function more effectively than their sole administration.	Olive oil is not expected to induce better effects as omega-3 FA. Olive oil acts as a good comparator because of similarity in taste, composition, consistency, and color. Exergame training can improve brain structure and function, but on a lower level as compared to the experimental group.
3. What materials?	Participants receive bottles including the fish oil, measuring cups, and a NS diary to record adherence.	Participants receive bottles including the olive oil, measuring cups, and a NS diary to record adherence.
	On pressure sensitive dance plates, participants perform whole body movements driven by VGs presented on a frontal screen.	
4. What procedure?	The participants take the NS daily. The PE includes six different VGs whereas each VG adapts the difficulty level to the participant's abilities. Each exergame is designed to train different executive and physical functions. One 30 min-training includes one session of each VG (4 min) with short breaks (~1 min) for game change.	
5. Who provides?	Investigators instructed to NS and exergame training.	
6. How?	For the NS, both intervention groups receive initial instruction about intake, duration, and dosage by an exercised investigator. The PE is performed in small groups supervised by experienced investigators (master students in human movement sciences at ETH Zurich).	
7. Where?	The participants take the NS at home. The PE is performed in training rooms at ETH Hönggerberg (Switzerland).	
8. When and how much?	For 26 weeks, the participant takes 13.5 ml of the NS daily. After 16 weeks, the participants continue with the NS and start with the PE. The PE takes place three times per week (30 min) for 10 weeks.	
9. Tailoring	The PE is tailored to the abilities of each individual participant by the integrated progression algorithm. If a participant gets better/worse in performance, the VG automatically adapts and becomes more difficult/easier.	

*FA, fatty acid; NS, nutritional supplementation; PE, physical exercise; VG, video game*.

#### Nutritional supplementation

Participants randomized to the experimental group take a liquid (oily consistency) fish oil (San Omega AS, Akersbakken 35B, NO-0172 Oslo). Participants randomized to the control group take olive oil as placebo (Oro del Desierto, Ctra. Nacional 340, 04200 Tabernas, Almeria, Spain). The reasons for choosing olive oil as comparator are the similarity of taste, composition, consistency, and color. Thus, olive oil is the most commonly used placebo for omega-FA studies (Miller et al., 2014).

Over 26 weeks, the participants take a daily amount of 13.5 ml of fish oil, including 2.9 g of omega-3 FA, or 13.5 ml of olive oil. The first 16 weeks, the participants take the NS with the aim of reaching a steady state (Katan et al., 1997; Arterburn et al., 2006; Stonehouse, 2014). A review on omega-3 FA suggests that a duration of 16 weeks is needed to account for potential interaction effects of gender and age (Stonehouse, 2014). Moreover, the time frame of 16 weeks is the minimum time needed for red blood cells to reach a steady state (Katan et al., 1997; Arterburn et al., 2006). The duration and dosage of the omega-3 FA was based on findings of previous studies. Two studies showed no detectable cognitive benefits when considering an intake of 0.7 g for 24 months (Dangour et al., 2010) or 1.8 g for 26 weeks (van de Rest et al., 2014), respectively. A possible explanation might be that the dosage level is more important than the time frame. Witte et al. (2014) utilized 2.2 g for 26 weeks and achieved a significant increase of EF and beneficial effects in white-matter microstructure integrity and on gray-matter volume (Witte et al., 2014). A review identified low to moderate side effects in form of gastrointestinal upset, fishy aftertaste, worsening glycemia, and rise in low density lipoprotein cholesterol for 1–3 g/d of omega-3 FA (Kris-Etherton et al., 2003).

At home, the NS can be taken undiluted or can be added to food (e.g., salads) or drinks. At intervention start, the participants receive bottles including the NS, measuring cups (13.5 ml), and oral as well as written information about duration, dosage, and intake. To check for intake adherence, the participants are supplied with a NS diary including week days and daytime.

#### Exergame training

On a pressure-sensitive dance plate (Impact Dance Platform, 87.5 × 87.5 × 2.5 cm, Positive Gaming BV, BZ Haarlem, Nederland), the participants perform specific whole body movements triggered by a video game (VG) presented on a frontal screen. The dance pad is connected by USB to a desktop computer and with symbols projected on a wall using a beamer. Electronic sensors in the dance pad detect position and timing information that are used to provide participants with real-time visual and auditory feedback. Through foot pushes on the plate arrows (right, left, top, and bottom), the participants interact with the game. The VGs (dividat, Schindellegi, Switzerland) are designed to train different aspects of EFs (divided attention, working memory, inhibition, and shifting) and physical functions. The exergame training allows the implementation of training principles as described in the paper of Healy et al. (2014) a feedback system to facilitate training, individual levels of difficulty according to individual skills and abilities, adjustable task difficulty to facilitate retention, and variability of training to enhance task transfer. Additionally, the FITT training principles are implemented; Frequency: three times per week, Intensity: individually adapted VG (allowing training progression), Type: combination of cognitive and motor training, and Time: 30 min training sessions.

After 16 weeks of NS intake, all participants start to perform the exergame training that lasts 10 weeks. The participants train for 30 min, three times per week. Based on the results of a meta-analysis, the time frame of 10 weeks was chosen in terms of an expected effect size of 0.478 (Colcombe and Kramer, 2003). The time frame and training intensity were, furthermore, based upon studies illustrating positive training effects in older adults performing a VG on a dance plate (Pichierri et al., 2012a,b). Training includes one session of each VG (4 min) in a pre-defined order and short breaks (~1 min) for game change. In training rooms (ETH Hönggerberg, Switzerland), the participants perform their exercises in small groups supervised by experienced investigators. To control for adherence, the participants receive a training plan including dates and time schedule. Furthermore, the investigators control adherence by a training adherence checklist. A previous trial testing the effects of similar games in older adults showed that this program will effect on EFs (Schättin et al., 2016).

### Staff eligibility

All involved investigators received training for data collection and handling in accordance with the study measurement protocols. Additionally, the investigators were instructed on how to prepare the participants for correct maintenance of the diary and NS intake. Furthermore, the investigators guiding the PE got instructions about the handling of the game console, game software, and the training procedure. The trained investigators supervise PE to explain the VG (if needed) and to minimize the risk of falls.

### Outcomes

All measurements are performed at pre- and post-intervention. The primary and secondary outcomes are listed in Table 2.

**Table 2:** **Trial outcomes**.

**Assessment methodology**	**Outcomes**	**Indication**
**PRIMARY OUTCOME**		
TMS	Motor evoked potential (right M. tibialis anterior)	Excitability of neuronal system, indirect measure of synaptic plasticity (Voss et al., 2013)
EEG	Response-related potential	Neuronal activity
**SECONDARY OUTCOME**		
TAP	Reaction time	Cognitive functioning
Gait	Temporal and spatial parameters	Motor functioning
	DTC	Cognitive cost
Blood sample	FA levels	Indicator for NS
**OTHER OUTCOMES**		
Short FES-I	Points (7–28)	“Concern” about falling
MMSE	Points (0–30)	Mental status
GDS	Points (0–15)	Depression status

*DTC, dual-task cost; EEG, electroencephalography; FES-I, falls efficacy scale international; GDS, geriatric depression scale; MMSE, mini mental state examination; NS, nutritional supplementation; TAP, test for attentional performance; TMS, transcranial magnetic stimulation*.

#### Transcranial magnetic stimulation

Participants sit comfortably on an adjustable chair with hip, knee, and ankle joint angles of 100°, 120°, and 90°, respectively. Given the symmetrical nature of transcranial magnetic stimulation (TMS)-related measurements of the lower limb, only the dominant side is assessed (Cacchio et al., 2009). Cortical stimulation is applied by means of a TMS stimulator MAGSTIM 200 (Magstim Company Ltd., Whitland, Dyfed, UK) with a “figure of eight” coil placed over the cortical motor area to stimulate the right M. tibialis anterior (TA). In healthy participants, TMS-related measurements of the TA are reliable (Cacchio et al., 2009).

Muscle activity is recorded by Telemyo DTS (Noraxon, Cologne, Germany). Before the measurement, the skin of the shank is shaved (if needed) and prepared with an abrasive gel (OneStep AbrasivPlus, H+H Medizinprodukte, Münster, Germany). The electrodes (Ambu® Blue Sensor N, Cambridgeshire, UK) are placed with an inter-distance of two cm on the muscle belly of the right TA. The muscle belly is defined through contraction of the TA.

In the first step, the participants are handed a bathing cap that fits tightly on the head. On the top of the cap, a grid is drawn using the vertex as initial position. The vertex is determined as half distance from nasion to inion and half distance from right to left pre-tragus. As previously suggested, the optimal activation of the TA is obtained if the coil is placed parallel to and approximately 0.5–1.0 cm lateral to the midline and its mid-point is aligned anterior-posteriorly against the vertex (Cz) (Devanne et al., 1997). To maintain consistent coil positioning across sessions, detailed distance recordings are made from the nasion, inion, and bilateral pre-tragus to the vertex. In the second step, the optimal stimulation point is assessed (hotspot). The hotspot corresponds, on the grid, to the lowest motor threshold that evokes a motor evoked potential (MEP) response (Rossini et al., 1994). The third step involves determination of the resting motor threshold (RMT) defined as the lowest intensity of magnetic stimulation required to evoke MEPs of 50 μV in peak-to-peak amplitude in at least 6 of 10 consecutive trials (Rossini et al., 1994). In the fourth step, a recruitment curve (RC) of increasing intensities of 10% steps is obtained in 10 trials per step. The stimuli intensities from 90% RMT to 140% RMT are applied in a random order. The interval between the stimuli is 7 s with a 20% variance to avoid familiarization. The analysis of peak-to-peak amplitude (MEP) and RC will be performed in Matlab™ for Windows (Mathworks Inc., Natick, MA, USA).

#### Electroencephalography

For electroencephalography (EEG) measurement, the participants wear a 20-channel dry-electrodes Enobio device (Neuroelectrics, Barcelona, Spain; Ruffini et al., 2006, 2007). The EEG system records and visualizes 24 bit EEG data at 500 Hz. The device sends the data via wireless connections to a personal computer where data can be monitored in real-time. During the EEG recording, the participants perform a Go/No-Go task including the suppression of a response in the presence of irrelevant stimuli. The stimuli presentation of the Go/No-Go task stems from the Test for Attentional Performance (TAP). The task is presented on a personal computer screen in front of the participants for about 10 min (five times 2 min). On a keyboard, the participants have to push a predefined button when the relevant stimuli appear. One investigator records the right and wrong event-related responses of the participants comparing the stimuli of the Go/No-Go task and the trigger appearing on the EEG screen. At the time point of clicking, a trigger is recorded and integrated into the EEG data recording. The trigger time points will be used for further analysis of the EEG data including response-related potentials (RRP). The analysis will be performed in Matlab™ for Windows (Mathworks Inc., Natick, MA, USA).

#### Test for attentional performance

The TAP (D-TAP 2.3 VL, PSYTEST, Psychologische Testsysteme, Herzogenrath, Germany) was initially developed to assess deficits in attention. The TAP is a valid test with the subtests measuring different and statistically independent attentional aspects (Zimmermann and Fimm, 2002). On a frontal screen, the participants see each test running on a personal computer. A button, placed in front of the participants, is used to record the reaction time and failure rate of the participants. Before the main test starts, the participants perform a pre-test to clarify the procedure and to minimize possible learning effects. The participants execute two tests: (1) Working memory (5 min): The participant has to compare presented double-digit numbers on the screen with previously exposed double-digit numbers. By pressing the button, the participants indicate the repetition of a number within a short interval; and (2) Divided attention (3.25 min): This subtest consists of visual and acoustic signals presented in an asynchronous way. In a 4 × 4 matrix, the visual task consisting of crosses appearing in a random configuration. The acoustic part consists of low and high beeps playing in a regular sequence. The participant has to detect whether the cross forms the corners of a square or whether the beeps have an irregularity in their sequence.

#### Gait analysis

Temporal (time) and spatial (distance) gait parameters are measured with the Physilog (Gait up Sàrl, Lausanne, Switzerland) via wearable standalone movement sensors (50 × 37 × 9.2 mm, 19 g, anatomical curved shape) containing inertial sensors. A button on the sensors allows the start and stop of measurement. A micro-USB port allows data transfer to the personal computer for further analysis of gait performance data. Physilog provides objective, quantitative, and valid assessment of gait movement (Aminian et al., 1999; Dubost et al., 2006; de Bruin et al., 2007; Mariani et al., 2012). The sensors are fixed with elastic straps at the right and left forefoot of the participants for flat over ground gait analysis over a distance of 10 m. Participants perform a single-task condition (preferred walking) and a dual-task condition, i.e., preferred walking whilst counting backwards in sevens from a random given number. The participants are instructed to position themselves at the beginning of the walkway and are asked to walk with their comfortable speed to the end of the walkway. Thereafter, the participants are asked to perform the same walking task while counting. For counting, the participants get a random number between 200 and 250 at the start. The instructions are standardized as follows: (1) “Walk with your comfortable speed right to the end of the walkway.” (2) “Walk with your comfortable speed right to the end of the walkway counting backwards from [random number between 200 and 250].” The participants have to count loud and don't stop walking; otherwise, the trial is recorded as failure. Instructions are given that no one task should be prioritized over the other. Assistive devices like canes, crutches or walking frames can be used if necessary. Each tested condition is repeated three successful times to obtain representative samples and the means of the three successful trials will be used for further data analysis. For each participant the relative dual-task costs (DTC) of walking, as percentage of loss relative to the single-task walking performance, according to the formula DTC [%] = 100^*^ (single-task score-dual-task score)/ single-task score (McDowd, 1986) will be calculated.

#### Blood sample

Venous blood samples are collected by a qualified investigator and stored in 2.7 ml EDTA tubes (S-Monovette, K3 EDTA, 75 × 13 mm, Sarstedt, Germany). Blood samples are taken to analyze FA values in erythrocytes. This direct method for FA analysis is reliable and accurate with Limits of Detection of the FAs profiles ranging between 0.23 and 3.19 μg (Rodriguez-Palmero et al., 1998). Pre- and post-dosage FA values will be assessed and compared with reference values previously reported (Superko et al., 2013). The FA parameters will be analyzed by Omegametrix GmbH (Martinsired, Germany). The laboratory meets the strict criteria of the quality standard DIN ISO 15189.

#### Mini mental state examination

The Mini Mental State Examination (MMSE) is a reliable and valid test to quantitatively estimate the severity of cognitive impairment (Folstein et al., 1975; Tombaugh and McIntyre, 1992). The 30 questions of the MMSE are categorized into seven categories: (1) orientation to time, (2) orientation to place, (3) registration of three words, (4) attention and calculation, (5) recall of three words, (6) language, and (7) visual construction. An investigator performs the test with the participants by giving zero points or one point for incorrect or correct answer, respectively.

#### Geriatric depression scale

The Geriatric Depression Scale (GDS) is a self-report questionnaire to identify depression in older adults (Yesavage et al., 1983). The GDS is a valid and reliable depression screening (Yesavage et al., 1983). The short form has 15 questions focusing on worries of the participants, and the way they conceive and interpret their quality of life (Yesavage and Sheikh, 1986). The questionnaire can be answered in a yes/no response.

### Data collection

All consenting participants received a case report form (CRF) to ensure that eligibility criteria are met and to ensure all measurements are performed. The CRF includes a confirmation that participants read the information sheet and signed the informed consent. Furthermore, the list of inclusion and exclusion criteria is included to confirm the eligibility of the participants. For the measurements, the steps are cross-checked for digital recording data (TMS, EEG, TAP, and gait), results are noted (short FES-I, GDS, MMSE) and blood taking is confirmed. The data from TMS, EEG, TAP, and gait are stored on a personal computer for further analysis. Any digital data, blood samples, questionnaires, and CRFs are coded with the individuals' ID.

### Sample size

To avoid a type I or II error, the power calculation was based on a study examining the effect of omega-3 FA on EEG frequency band distribution during a sustained attention test (Fontani et al., 2005). The aforementioned study was used for sample size calculation because so far there exists no study that examined the influence of exergame training in combination with omega-3 FA on neuronal systems using TMS or EEG methodology. Due to the values of EEG frequency band distribution during sustained attention tests (with values K + K = 27.70 ± 5.2; K + K = 31.49 ± 8.6), an estimated sample size of 24 participants would result in 80% power at an alpha-level of 0.05 for this parameter. To account and compensate for expected drop-outs, the study includes 30 participants in each group. Drop-outs are expected because of the long study duration of 26 weeks, the age of the participants, and numbers are based on previous reports on adherence of non-institutionalized older adults to exercise programs (Nyman and Victor, 2012).

### Statistics

The data analysis will be performed at the end of intervention including the measurement values from pre- and post-intervention measurements. Data will be tested for normal distribution using Shapiro-Wilk test and Q-Q-plots. A 2 × 2-ANOVA will be used with normally distributed data, the non-parametric equivalent for data not fulfilling assumptions of normal distribution. The test will be used to compare the two interventions over time (from pre- to post-measurement) on changes on the main dependent variables MEP, RRP, RT of cognitive tasks, spatio-temporal gait parameters, DTC, and FA levels. In addition to statistical significance testing effect size calculation (*r* = Z/√N) will be performed. The participants' FA blood levels, demographic, and health information will be examined in relation to the outcome measures in order to interpret the results in context. All statistical procedures will be conducted with the IBM Statistical Package for the Social Science software package. A probability level of *p* < 0.05 will be considered to be statistically significant.

## Stepwise procedure

**Figure 1:**
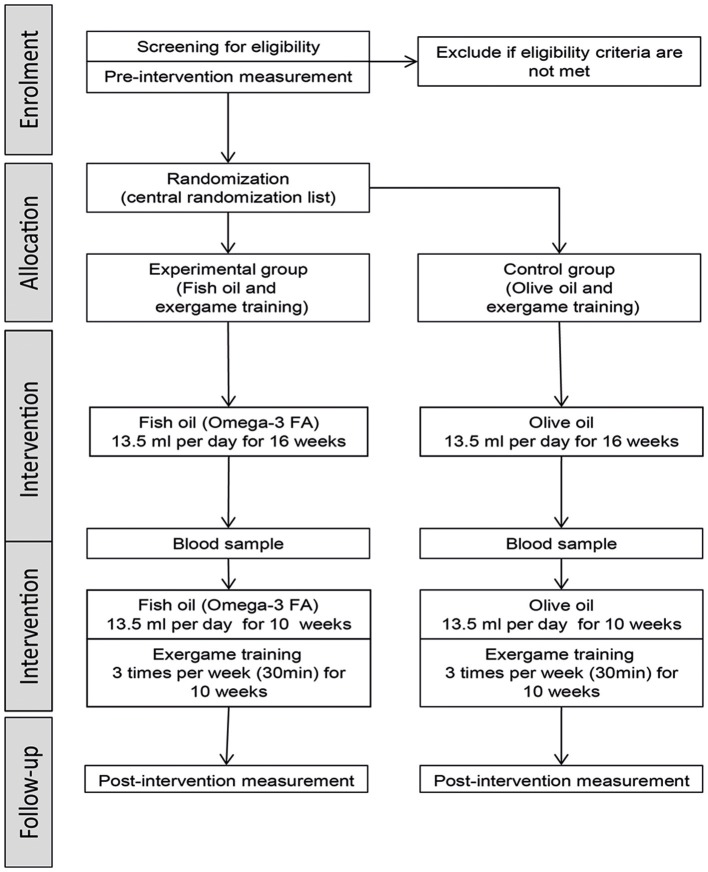
**Flow chart of study procedure. FA: fatty acid**.

The stages of the study procedure are illustrated in Figure 1. The study includes two measurement time points (pre- and post-intervention) which are performed in a laboratory at the ETH Zurich (Hönggerberg, Switzerland). The two data collection sessions are performed by treatment-blinded investigators including the following assessments: TMS measurement, EEG measurement, TAP performance, gait performance, and questionnaires (short FES-I, MMSE, and GDS). The pre-measurement consists of a screening and measurement part. The screening part contains data of the MMSE, GDS, and health questionnaire (including questions about physical impairments, medical history, anthropometric data, and physical activity level) and is used to determine eligibility for the measurement part and study participation, respectively. The measurements for each session take about 2 h and are conducted to determine the effects of the interventions on brain structure and function. The pre-intervention measurement is planned to be performed in the week before the intervention starts. The post-intervention measurement is planned for the first week after the intervention, while the possibility consists to postpone the measurement by one week. In addition, blood samples are taken at pre-intervention measurement, after 16 weeks of NS intervention, and at post-intervention measurement.

The intervention starts on Monday of the week following the pre-intervention measurement and lasts 26 weeks. Each day for 26 weeks, the participants have to take the same amount of NS regularly at home, while the intake time point should be as consistent as possible. The investigators can reproduce the intake using the individual intake protocol of each participant. After 16 weeks, the participants start with the exergame training lasting for 10 weeks. In small groups, each participant trains three times per week for 30 min. The training time points are individualized for each participant and may also be variable from week to week for individual participants. Each participant has to attend at least 70% of training to be considered for (per protocol) analysis.

## Anticipated results

The results of this study can be influenced by several factors. One might be the adherence to regular intake of the NS. As countermeasures an intake protocol supports the participant and helps the investigator to control the intake. Blood sample analysis is an additional control measure. Moreover, the training attendance is checked by the investigators, so that each participant reaches at least 70% attendance. However, a higher drop-out rate may be expected because of the long trial duration. The aforementioned sample size calculation included the calculated 25 participants and five additional participants for each intervention group because of the expected drop-outs. Moreover, TMS is used in this study as an indirect proxy measure of synaptic plasticity. Based on the results of this study future studies should focus on more direct measures of synaptic plasticity by using either magnetic resonance tomography (Alvarez-Salvado et al., 2014) or positron emissions tomography scans (Magistretti, 2006) for comparing the brain scans before and after the intervention.

The aim of this study is to investigate the neuronal effects of an intervention that combines exergame training with omega-3 FA supplementation. Several animal studies combined omega-3 FA and PE focusing on the neuronal effects and the possible underlying mechanism. One study concluded that omega-3 FA interacts with PE in improving the axonal growth, synaptic plasticity, and cognitive function of the adult rat brain (Chytrova et al., 2010). Omega-3 FA and PE might act both on the energy metabolism of hypothalamus and hippocampus thereby influencing brain plasticity and cognitive function (Gomez-Pinilla and Ying, 2010). Moreover, the combination may increase the level of brain-derived neurotrophic factor resulting in the activation of CREB and synapsin I (Wu et al., 2008). The activated metabolic pathway supports neuroplasticity and cognition. So far, no comparable human study exists that combines PE, especially exergame training, and omega-3 FA. The trial design is based on the results of the aforementioned animal studies and of the sole administration studies of either exergame training or omega-3 FA for older participants focusing on the brain. An exergame intervention was chosen because a combination of cognitive and motor training is expected to have positive effects on the elderly brain (Bamidis et al., 2014; Law et al., 2014; Schättin et al., 2016) and is able to ameliorate EFs in older adults (Eggenberger et al., 2016; Schättin et al., 2016). Motor training builds new synaptic connections while the cognitive part supports the preservation of the new build structure. In aging humans, PE can strengthen neuronal structure, synaptic plasticity, and transmission as well as cognitive function (Cai et al., 2014). PE might trigger molecular and cellular mechanisms supporting brain plasticity (Cotman et al., 2007). Furthermore, video game-based training serves as a powerful tool to train cognitive abilities (Zelinski and Reyes, 2009; Kueider et al., 2012), including attention and EFs (Jobe et al., 2001) as well as to evaluate functioning of underlying neuronal mechanisms explaining cognitive control (Anguera et al., 2013). In normal aging humans, omega-3 FA might improve cognition (Janssen and Kiliaan, 2014) and ameliorate brain deterioration through the positive effects on brain structure, function, and cerebral blood flow (Haast and Kiliaan, 2015). A recent randomized-controlled trial showed that omega-3 FA had positive effects on EF, white matter microstructure integrity, gray matter volume, and vascular parameters (Witte et al., 2014). Both interventions, exergame training and omega-3 FA, are believed to act on the same (metabolic) brain pathways and, therefore, complement each other (Gómez-Pinilla, 2008). The brain gets trained during the exergame training and the omega-3 FA might, in this case, provide the needed substance and energy to build up new structures and to support metabolic pathways. An interaction is created that might be more effective for brain structure and function compared to the sole administration of an individual intervention component.

## Author contributions

AS and EDB developed the research question. AS developed the study design and measurements protocol while EDB acted as methodological council. EDB edited and revised the study protocol from AS. Both authors have read and approved the final manuscript.

## Funding

This article was supported by the ETH Foundation through ETH Research Grant ETH-17 13-2.

### Conflict of interest statement

The authors declare that the research was conducted in the absence of any commercial or financial relationships that could be construed as a potential conflict of interest.

## Abbreviations

CREB: cAMP response element-binding protein
CRF: case report from
DTC: dual task cost
EDTA: ethylenediaminetetraacetic acid
EEG: electroencephalography
EF: executive function
FA: fatty acid
FES-I: falls efficacy scale international
FITT: frequency, intensity, type and time
GDS: geriatric depression scale
ID: identification
LCPUFA: long chain polyunsaturated fatty acid
MEP: motor evoked potential
MMSE: mini mental state examination
NS: nutritional supplementation
PE: physical exercise
PFC: prefrontal cortex
RC: recruitment curve
RMT: resting motor threshold
RRP: response-related potential
TAP: test for attentional performance
TMS: transcranial magnetic stimulation
VG: video game.

## References

[B1] Alvarez-Salvado E. Pallarés V. Moreno A. Canals S. (2014). Functional MRI of long-term potentiation: imaging network plasticity. Philos. Trans. R. Soc. Lond. B Biol. Sci. 369:20130152. 10.1098/rstb.2013.0152 24298154 PMC3843884

[B2] Aminian K. Robert P. Buchser E. E. Rutschmann B. Hayoz D. Depairon M. (1999). Physical activity monitoring based on accelerometry: validation and comparison with video observation. Med. Biol. Eng. Comput. 37, 304–308. 10505379 10.1007/BF02513304

[B3] Anguera J. A. Boccanfuso J. Rintoul J. L. Al-Hashimi O. Faraji F. Janowich J.. (2013). Video game training enhances cognitive control in older adults. Nature 501, 97–101. 10.1038/nature12486 24005416 PMC3983066

[B4] Arterburn L. M. Hall E. B. Oken H. (2006). Distribution, interconversion, and dose response of n-3 fatty acids in humans. Am. J. Clin. Nutr. 83(6 Suppl), 1467S-1476S. 16841856 10.1093/ajcn/83.6.1467S

[B5] Bäckman L. Nyberg L. Lindenberger U. Li S. C. Farde L. (2006). The correlative triad among aging, dopamine, and cognition: current status and future prospects. Neurosci. Biobehav. Rev. 30, 791–807. 10.1016/j.neubiorev.2006.06.005 16901542

[B6] Bamidis P. D. Vivas A. Styliadis C. Frantzidis C. Klados M. Schlee W.. (2014). A review of physical and cognitive interventions in aging. Neurosci. Biobehav. Rev. 44, 206–220. 10.1016/j.neubiorev.2014.03.019 24705268

[B7] Banich M. T. Mackiewicz K. L. Depue B. E. Whitmer A. J. Miller G. A. Heller W. (2009). Cognitive control mechanisms, emotion and memory: a neural perspective with implications for psychopathology. Neurosci. Biobehav. Rev. 33, 613–630. 10.1016/j.neubiorev.2008.09.010 18948135 PMC2865433

[B8] Boutron I. Moher D. Altman D. G. Schulz K. F. Ravaud P. (2008). Extending the CONSORT statement to randomized trials of nonpharmacologic treatment: explanation and elaboration. Ann. Intern. Med. 148, 295–309. 10.7326/0003-4819-148-4-200802190-00008 18283207

[B9] Brickman A. M. Zimmerman M. E. Paul R. H. Grieve S. M. Tate D. F. Cohen R. A.. (2006). Regional white matter and neuropsychological functioning across the adult lifespan. Biol. Psychiatry 60, 444–453. 10.1016/j.biopsych.2006.01.011 16616725

[B10] Cacchio A. Cimini N. Alosi P. Santilli V. Marrelli A. (2009). Reliability of transcranial magnetic stimulation-related measurements of tibialis anterior muscle in healthy subjects. Clin. Neurophysiol. 120, 414–419. 10.1016/j.clinph.2008.11.019 19135412

[B11] Cai L. Chan J. S. Yan J. H. Peng K. (2014). Brain plasticity and motor practice in cognitive aging. Front. Aging Neurosci. 6:31. 10.3389/fnagi.2014.00031 24653695 PMC3947993

[B12] Chan A.-W. Tetzlaff J. M. Altman D. G. Laupacis A. Gøtzsche P. C. Krleža-Jerić K.. (2013). SPIRIT 2013 statement: defining standard protocol items for clinical trials. Ann. Intern. Med. 158, 200–207. 10.7326/0003-4819-158-3-201302050-00583 23295957 PMC5114123

[B13] Chytrova G. Ying Z. Gomez-Pinilla F. (2010). Exercise contributes to the effects of DHA dietary supplementation by acting on membrane-related synaptic systems. Brain Res. 1341, 32–40. 10.1016/j.brainres.2009.05.018 19446534 PMC2884051

[B14] Colcombe S. J. Erickson K. I. Raz N. Webb A. G. Cohen N. J. McAuley E.. (2003). Aerobic fitness reduces brain tissue loss in aging humans. J. Gerontol. A Biol. Sci. Med. Sci. 58, 176–180. 10.1093/gerona/58.2.M176 12586857

[B15] Colcombe S. Kramer A. F. (2003). Fitness effects on the cognitive function of older adults: a meta-analytic study. Psychol. Sci. 14, 125–130. 10.1111/1467-9280.t01-1-01430 12661673

[B16] Cotman C. W. Berchtold N. C. Christie L. A. (2007). Exercise builds brain health: key roles of growth factor cascades and inflammation. Trends Neurosci. 30, 464–472. 10.1016/j.tins.2007.06.011 17765329

[B17] Dangour A. D. Allen E. Elbourne D. Fasey N. Fletcher A. E. Hardy P.. (2010). Effect of 2-y n-3 long-chain polyunsaturated fatty acid supplementation on cognitive function in older people: a randomized, double-blind, controlled trial. Am. J. Clin. Nutr. 91, 1725–1732. 10.3945/ajcn.2009.29121 20410089

[B18] Davis S. W. Dennis N. A. Buchler N. G. White L. E. Madden D. J. Cabeza R. (2009). Assessing the effects of age on long white matter tracts using diffusion tensor tractography. NeuroImage 46, 530–541. 10.1016/j.neuroimage.2009.01.068 19385018 PMC2775533

[B19] de Bruin E. D. Najafi B. Murer K. Uebelhart D. Aminian K. (2007). Quantification of everyday motor function in a geriatric population. J. Rehabil. Res. Dev. 44, 417–428. 10.1682/JRRD.2006.01.0003 18247238

[B20] de Bruin E. D. Reith A. Dorflinger M. Murer K. (2011). Feasibility of strength-balance training extended with computer game dancing in older people; does it affect dual task costs of walking? J. Nov. Physiother. 1:104 10.4172/2165-7025.1000104

[B21] de Bruin E. D. Schmidt A. (2010). Walking behaviour of healthy elderly: attention should be paid. Behav. Brain Funct. 6:59. 10.1186/1744-9081-6-59 20939911 PMC2959004

[B22] de Bruin E. D. van Het Reve E. Murer K. (2013). A randomized controlled pilot study assessing the feasibility of combined motor–cognitive training and its effect on gait characteristics in the elderly. Clin. Rehabil. 27, 215–225. 10.1177/0269215512453352 22865831

[B23] DeLong M. R. (2000). Functional and pathophysiological models of the basal ganglia: therapeutic implications. Rinsho Shinkeigaku 40:1184. 11464452

[B24] Devanne H. Lavoie B. A. Capaday C. (1997). Input-output properties and gain changes in the human corticospinal pathway. Exp. Brain Res. 114, 329–338. 10.1007/PL00005641 9166922

[B25] Dubost V. Kressig R. W. Gonthier R. Herrmann F. R. Aminian K. Najafi B.. (2006). Relationships between dual-task related changes in stride velocity and stride time variability in healthy older adults. Hum. Mov. Sci. 25, 372–382. 10.1016/j.humov.2006.03.004 16714067

[B26] Eggenberger P. Wolf M. Schumann M. de Bruin E. D. (2016). Exergame and balance training modulate prefrontal brain activity during walking and enhance executive function in older adults. Front. Aging Neurosci. 8:66. 10.3389/fnagi.2016.00066 27148041 PMC4828439

[B27] Fjell A. M. Walhovd K. B. (2010). Structural brain changes in aging: courses, causes and cognitive consequences. Rev. Neurosci. 21, 187–222. 10.1515/REVNEURO.2010.21.3.187 20879692

[B28] Folstein M. F. Folstein S. E. McHugh P. R. (1975). “Mini-mental state”. A practical method for grading the cognitive state of patients for the clinician. J. Psychiatr. Res. 12, 189–198. 10.1016/0022-3956(75)90026-6 1202204

[B29] Fontani G. Corradeschi F. Felici A. Alfatti F. Migliorini S. Lodi L. (2005). Cognitive and physiological effects of Omega-3 polyunsaturated fatty acid supplementation in healthy subjects. Eur. J. Clin. Invest. 35, 691–699. 10.1111/j.1365-2362.2005.01570.x 16269019

[B30] Gómez-Pinilla F. (2008). Brain foods: the effects of nutrients on brain function. Nat. Rev. Neurosci. 9, 568–578. 10.1038/nrn2421 18568016 PMC2805706

[B31] Gomez-Pinilla F. Ying Z. (2010). Differential effects of exercise and dietary docosahexaenoic acid on molecular systems associated with control of allostasis in the hypothalamus and hippocampus. Neuroscience 168, 130–137. 10.1016/j.neuroscience.2010.02.070 20303394 PMC3225187

[B32] Green C. S. Bavelier D. (2008). Exercising your brain: a review of human brain plasticity and training-induced learning. Psychol. Aging 23, 692–701. 10.1037/a0014345 19140641 PMC2896818

[B33] Gunning-Dixon F. M. Brickman A. M. Cheng J. C. Alexopoulos G. S. (2009). Aging of cerebral white matter: a review of MRI findings. Int. J. Geriatr. Psychiatry 24, 109–117. 10.1002/gps.2087 18637641 PMC2631089

[B34] Gunning-Dixon F. M. Raz N. (2003). Neuroanatomical correlates of selected executive functions in middle-aged and older adults: a prospective MRI study. Neuropsychologia 41, 1929–1941. 10.1016/S0028-3932(03)00129-5 14572526

[B35] Haast R. A. Kiliaan A. J. (2015). Impact of fatty acids on brain circulation, structure and function. Prostaglandins Leukot. Essent. Fatty Acids 92, 3–14. 10.1016/j.plefa.2014.01.002 24485516

[B36] Hausdorff J. M. Yogev G. Springer S. Simon E. S. Giladi N. (2005). Walking is more like catching than tapping: gait in the elderly as a complex cognitive task. Exp. Brain Res. 164, 541–548. 10.1007/s00221-005-2280-3 15864565

[B37] Healy A. F. Kole J. A. Bourne L. E. Jr. (2014). Training principles to advance expertise. Front. Psychol. 5:131. 10.3389/fpsyg.2014.00131 24600425 PMC3928555

[B38] Hoffmann T. C. Glasziou P. P. Boutron I. Milne R. Perera R. Moher D.. (2014). Better reporting of interventions: template for intervention description and replication (TIDieR) checklist and guide. BMJ 348:g1687. 10.1136/bmj.g1687 24609605

[B39] Holtzer R. Verghese J. Xue X. Lipton R. B. (2006). Cognitive processes related to gait velocity: results from the Einstein Aging Study. Neuropsychology 20, 215–223. 10.1037/0894-4105.20.2.215 16594782

[B40] Janssen C. I. Kiliaan A. J. (2014). Long-chain polyunsaturated fatty acids (LCPUFA) from genesis to senescence: the influence of LCPUFA on neural development, aging, and neurodegeneration. Prog. Lipid Res. 53, 1–17. 10.1016/j.plipres.2013.10.002 24334113

[B41] Jobe J. B. Smith D. M. Ball K. Tennstedt S. L. Marsiske M. Willis S. L.. (2001). ACTIVE: a cognitive intervention trial to promote independence in older adults. Control. Clin. Trials 22, 453–479. 10.1016/S0197-2456(01)00139-8 11514044 PMC2916177

[B42] Katan M. B. Deslypere J. P. van Birgelen A. P. Penders M. Zegwaard M. (1997). Kinetics of the incorporation of dietary fatty acids into serum cholesteryl esters, erythrocyte membranes, and adipose tissue: an 18-month controlled study. J. Lipid Res. 38, 2012–2022. 9374124

[B43] Kris-Etherton P. M. Harris W. S. Appel L. J. Nutrition C. (2003). Fish consumption, fish oil, omega-3 fatty acids, and cardiovascular disease. Arterioscler. Thromb. Vasc. Biol. 23, e20–e30. 10.1161/01.CIR.0000038493.65177.94 12588785

[B44] Kueider A. M. Parisi J. M. Gross A. L. Rebok G. W. (2012). Computerized cognitive training with older adults: a systematic review. PLoS ONE 7:e40588. 10.1371/journal.pone.0040588 22792378 PMC3394709

[B45] Law L. L. Barnett F. Yau M. K. Gray M. A. (2014). Effects of combined cognitive and exercise interventions on cognition in older adults with and without cognitive impairment: a systematic review. Ageing Res. Rev. 15, 61–75. 10.1016/j.arr.2014.02.008 24632497

[B46] Longstreth W. T. Jr. Manolio T. A. Arnold A. Burke G. L. Bryan N. Jungreis C. A.. (1996). Clinical correlates of white matter findings on cranial magnetic resonance imaging of 3301 elderly people. The cardiovascular health study. Stroke 27, 1274–1282. 10.1161/01.STR.27.8.1274 8711786

[B47] Magistretti P. J. (2006). Neuron-glia metabolic coupling and plasticity. J. Exp. Biol. 209(Pt 12), 2304–2311. 10.1242/jeb.02208 16731806

[B48] Mariani B. Rochat S. Büla C. J. Aminian K. (2012). Heel and toe clearance estimation for gait analysis using wireless inertial sensors. IEEE Trans. Biomed. Eng. 59, 3162–3168. 10.1109/TBME.2012.2216263 22955865

[B49] McDowd J. M. (1986). The effects of age and extended practice on divided attention performance. J. Gerontol. 41, 764–769. 10.1093/geronj/41.6.764 3772053

[B50] Miller P. E. Van Elswyk M. Alexander D. D. (2014). Long-chain omega-3 fatty acids eicosapentaenoic acid and docosahexaenoic acid and blood pressure: a meta-analysis of randomized controlled trials. Am. J. Hypertens. 27, 885–896. 10.1093/ajh/hpu024 24610882 PMC4054797

[B51] Mirelman A. Herman T. Brozgol M. Dorfman M. Sprecher E. Schweiger A.. (2012). Executive function and falls in older adults: new findings from a five-year prospective study link fall risk to cognition. PLoS ONE 7:e40297. 10.1371/journal.pone.0040297 22768271 PMC3386974

[B52] Nyman S. R. Victor C. R. (2012). Older people's participation in and engagement with falls prevention interventions in community settings: an augment to the Cochrane systematic review. Age Ageing 41, 16–23. 10.1093/ageing/afr103 21875865

[B53] Pichierri G. Coppe A. Lorenzetti S. Murer K. de Bruin E. D. (2012b). The effect of a cognitive-motor intervention on voluntary step execution under single and dual task conditions in older adults: a randomized controlled pilot study. Clin. Interv. Aging 7, 175–184. 10.2147/CIA.S32558 22865999 PMC3410679

[B54] Pichierri G. Murer K. de Bruin E. D. (2012a). A cognitive-motor intervention using a dance video game to enhance foot placement accuracy and gait under dual task conditions in older adults: a randomized controlled trial. BMC Geriatr. 12:74. 10.1186/1471-2318-12-74 23241332 PMC3538689

[B55] Rapport L. J. Hanks R. A. Millis S. R. Deshpande S. A. (1998). Executive functioning and predictors of falls in the rehabilitation setting. Arch. Phys. Med. Rehabil. 79, 629–633. 10.1016/S0003-9993(98)90035-1 9630140

[B56] Rodriguez-Palmero M. Lopez-Sabater M. C. Castellote-Bargallo A. I. De la Torre-Boronat M. C. Rivero-Urgell M. (1998). Comparison of two methods for the determination of fatty acid profiles in plasma and erythrocytes. J. Chromatogr. A 793, 435–440. 9474792

[B57] Rosano C. Studenski S. A. Aizenstein H. J. Boudreau R. M. Longstreth W. T. Jr. Newman A. B. (2012). Slower gait, slower information processing and smaller prefrontal area in older adults. Age Ageing 41, 58–64. 10.1093/ageing/afr113 21965414 PMC3234076

[B58] Rossini P. M. Barker A. T. Berardelli A. Caramia M. D. Caruso G. Cracco R. Q.. (1994). Non-invasive electrical and magnetic stimulation of the brain, spinal cord and roots: basic principles and procedures for routine clinical application. Report of an IFCN committee. Electroencephalogr. Clin. Neurophysiol. 91, 79–92. 10.1016/0013-4694(94)90029-9 7519144

[B59] Ruffini G. Dunne S. Farres E. Watts P. C. Mendoza E. Silva S. R. (eds.). (2006). Enobio-first tests of a dry electrophysiology electrode using carbon nanotubes, in Engineering in Medicine and Biology Societ, EMBS'06 28th Annual International Conference of the IEEE (New York, NY: IEEE). 10.1109/IEMBS.2006.259248 17946072

[B60] Ruffini G. Dunne S. Farrés E. Cester I. Watts P. C. Ravi S. (eds.). (2007). ENOBIO dry electrophysiology electrode; first human trial plus wireless electrode system, in Engineering in Medicine and Biology Society, 2007 EMBS 2007 29th Annual International Conference of the IEEE (Lyon: IEEE). 10.1109/IEMBS.2007.4353895 18003561

[B61] Schättin A. Arner R. Gennaro F. de Bruin E. (2016). Adaptations of prefrontal brain activity, executive functions, and gait in healthy elderly following exergame and balance training: a randomized-controlled study. Front. Aging Neurosci. 8:278 10.3389/fnagi.2016.00278 PMC5120107 27932975

[B62] Schättin A. Baur K. Stutz J. Wolf P. de Bruin E. D. (2016). Effects of physical exercise combined with nutritional supplements on aging brain related structures and functions: a systematic review. Front. Aging Neurosci. 8:161. 10.3389/fnagi.2016.00161 27458371 PMC4933713

[B63] Scherder E. Eggermont L. Visscher C. Scheltens P. Swaab D. (2011). Understanding higher level gait disturbances in mild dementia in order to improve rehabilitation: ‘last in-first out’. Neurosci. Biobehav. Rev. 35, 699–714. 10.1016/j.neubiorev.2010.08.009 20833200

[B64] Schliebs R. Arendt T. (2011). The cholinergic system in aging and neuronal degeneration. Behav. Brain Res. 221, 555–563. 10.1016/j.bbr.2010.11.058 21145918

[B65] Segev-Jacubovski O. Herman T. Yogev-Seligmann G. Mirelman A. Giladi N. Hausdorff J. M. (2011). The interplay between gait, falls and cognition: can cognitive therapy reduce fall risk? Expert Rev. Neurother. 11, 1057–1075. 10.1586/ern.11.69 21721921 PMC3163836

[B66] Sheridan P. L. Solomont J. Kowall N. Hausdorff J. M. (2003). Influence of executive function on locomotor function: divided attention increases gait variability in Alzheimer's disease. J. Am. Geriatr. Soc. 51, 1633–1637. 10.1046/j.1532-5415.2003.51516.x 14687395

[B67] Stonehouse W. (2014). Does consumption of LC Omega-3 PUFA enhance cognitive performance in healthy school-aged children and throughout adulthood? Evid. Clin. Trials Nutr. 6, 2730–2758. 10.3390/nu6072730 25054550 PMC4113767

[B68] Superko H. R. Superko S. M. Nasir K. Agatston A. Garrett B. C. (2013). Omega-3 fatty acid blood levels: clinical significance and controversy. Circulation 128, 2154–2161. 10.1161/CIRCULATIONAHA.113.002731 24190935

[B69] Thompson P. D. Nutt J. G. (2007). Higher level gait disorders. J. Neural Transm. 114, 1305–1307. 10.1007/s00702-007-0749-x 17497231

[B70] Tombaugh T. N. McIntyre N. J. (1992). The mini-mental state examination: a comprehensive review. J. Am. Geriatr. Soc. 40, 922–935. 10.1111/j.1532-5415.1992.tb01992.x 1512391

[B71] van de Rest O. van der Zwaluw N. L. Tieland M. Adam J. J. Hiddink G. J. van Loon L. J.. (2014). Effect of resistance-type exercise training with or without protein supplementation on cognitive functioning in frail and pre-frail elderly: secondary analysis of a randomized, double-blind, placebo-controlled trial. Mech. Ageing Dev. 136–137, 85–93. 10.1016/j.mad.2013.12.005 24374288

[B72] van Praag H. (2009). Exercise and the brain: something to chew on. Trends Neurosci. 32, 283–290. 10.1016/j.tins.2008.12.007 19349082 PMC2680508

[B73] Volkow N. D. Gur R. C. Wang G. J. Fowler J. S. Moberg P. J. Ding Y. S.. (1998). Association between decline in brain dopamine activity with age and cognitive and motor impairment in healthy individuals. Am. J. Psychiatry 155, 344–349. 10.1176/ajp.155.3.344 9501743

[B74] Voss M. W. Vivar C. Kramer A. F. van Praag H. (2013). Bridging animal and human models of exercise-induced brain plasticity. Trends Cogn. Sci. 17, 525–544. 10.1016/j.tics.2013.08.001 24029446 PMC4565723

[B75] Wang G. J. Volkow N. D. Fowler J. S. Ding Y. S. Logan J. Gatley S. J.. (1995). Comparison of two pet radioligands for imaging extrastriatal dopamine transporters in human brain. Life Sci. 57, PL187–PL191. 10.1016/0024-3205(95)02099-5 7564877

[B76] Wang Y. Chan G. L. Holden J. E. Dobko T. Mak E. Schulzer M.. (1998). Age-dependent decline of dopamine D1 receptors in human brain: a PET study. Synapse 30, 56–61. 10.1002/(SICI)1098-2396(199809)30:1<56::AID-SYN7>3.0.CO;2-J 9704881

[B77] Witte A. V. Kerti L. Hermannstädter H. M. Fiebach J. B. Schreiber S. J. Schuchardt J. P.. (2014). Long-chain omega-3 fatty acids improve brain function and structure in older adults. Cereb. Cortex 24, 3059–3068. 10.1093/cercor/bht163 23796946

[B78] Wu A. Ying Z. Gomez-Pinilla F. (2008). Docosahexaenoic acid dietary supplementation enhances the effects of exercise on synaptic plasticity and cognition. Neuroscience 155, 751–759. 10.1016/j.neuroscience.2008.05.061 18620024 PMC3208643

[B79] Yan J. H. Zhou C. L. (2009). Effects of motor practice on cognitive disorders in older adults. Eur. Rev. Aging Phys. A 6, 67–74. 10.1007/s11556-009-0049-6

[B80] Yesavage J. A. Brink T. L. Rose T. L. Lum O. Huang V. Adey M.. (1983). Development and validation of a geriatric depression screening scale: a preliminary report. J. Psychiatr. Res. 17, 37–49. 10.1016/0022-3956(82)90033-4 7183759

[B81] Yesavage J. A. Sheikh J. I. (1986). 9/Geriatric Depression Scale (GDS) recent evidence and development of a shorter violence. Clin. Gerontol. 5, 165–173. 10.1300/J018v05n01_09

[B82] Yogev-Seligmann G. Hausdorff J. M. Giladi N. (2008). The role of executive function and attention in gait. Mov. Disord. 23, 329–342. 10.1002/mds.21720 18058946 PMC2535903

[B83] Yuan P. Raz N. (2014). Prefrontal cortex and executive functions in healthy adults: a meta-analysis of structural neuroimaging studies. Neurosci. Biobehav. Rev. 42, 180–192. 10.1016/j.neubiorev.2014.02.005 24568942 PMC4011981

[B84] Zelinski E. M. Reyes R. (2009). Cognitive benefits of computer games for older adults. Gerontechnology 8, 220–235. 10.4017/gt.2009.08.04.004.00 25126043 PMC4130645

[B85] Zimmerman M. E. Brickman A. M. Paul R. H. Grieve S. M. Tate D. F. Gunstad J.. (2006). The relationship between frontal gray matter volume and cognition varies across the healthy adult lifespan. Am. J. Geriatr. Psychiatry 14, 823–833. 10.1097/01.JGP.0000238502.40963.ac 17001022

[B86] Zimmermann P. Fimm B. (2002). A test battery for attentional performance, in Applied Neurophyschology of Attention Theory, Diagnosis and Rehabilitation, eds Leclercq M. Zimmerman P. (London: Psychology Press), 110–151.

